# Supplementary material for: Splice-Junction-Based Mapping of Alternative Isoforms in the Human Proteome
Source: Cell Rep. Author manuscript; Available in PMC 2020 Jan 15. (PMC6961840; doi:10.1016/j.celrep.2019.11.026)

A

sp|Q8N4C8|MINK1_HUMAN|ENSG00000141503|RI1|5828|chr17|4889763|4890735|+2|r61|T4
AATAAGAGAAEAAGEGAAAAAGGHAGSAAGGGA q value: 0.00072141 Tr_novel:TRUE RefSeq_Novel:TRUE
Search result spec prec mz: 817.7142 Actual spec prec mz: 817.71411
Fragments matched per AA: 1.42 Proportion of top 20 peaks matched: 0.3

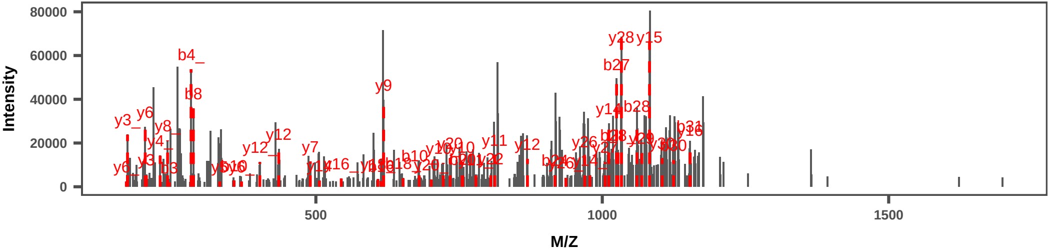

B

Scatterplot of predicted elution time
Fitting R2: 0.776
Novel peptide residual Z score: 4.29
Number of peptides: 2058

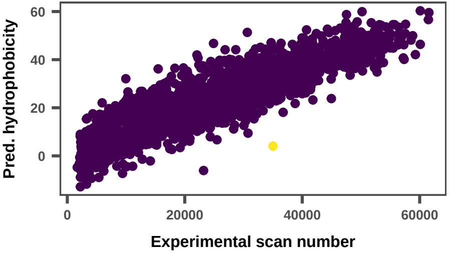

C

Distributions of residuals from best−fit line
of predicted RT vs Expt. scan number
Line: Z score of novel peptide
Z: 4.29

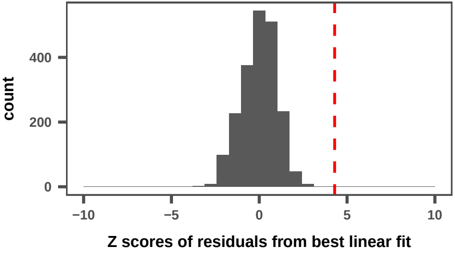

Supplement: 2 [file NIHMS1546469-supplement-2.zip › DF1/PXD010154/AdrenalGland/AdrenalGland_8_MINK1_AATAAGAGAAEAAGEGAAAAAGGHAGSAAGGGA.pdf]
